# Diagnostic performance of multi-branch coronary angiography-based index of microcirculatory resistance: a novel approach

**DOI:** 10.3389/fmed.2025.1490346

**Published:** 2025-01-17

**Authors:** Yongzhen Fan, Shuang Wang, Xinyong Cai, Xiaorong Hu, Jun Ma, Hongzhi Lan, Zhibing Lu

**Affiliations:** ^1^Institute of Myocardial Injury and Repair, Wuhan University, Wuhan, China; ^2^Department of Cardiology, Zhongnan Hospital, Wuhan University, Wuhan, Hebei Province, China; ^3^Department of Cardiovascular Ultrasound, Zhongnan Hospital of Wuhan University, Wuhan, China; ^4^Department of Cardiology, The First Affiliated Hospital of Nanchang Medical College, Nanchang, China; ^5^Shenzhen Raysightmed Co, Ltd, Shenzhen, China

**Keywords:** index of microcirculatory resistance (IMR), retrospective, coronary angiography-based IMR (CAG-IMR), computational fluid dynamics (CFD), coronary microvascular dysfunction (CMD)

## Abstract

**Background:**

Wire-based index of microcirculatory resistance (IMR) utilizing pressure wires and thermodilution techniques for the assessment of coronary microcirculatory function, presents challenges for clinical routine use due to its complexity, time-consuming, and costly. This study introduces a novel multi-branch and wire-free method for IMR calculation based on coronary angiography. The diagnostic performance of CAG-IMR is validated within a retrospective single-center investigation.

**Methods:**

In a retrospective single-center study, 139 patients with 201 vessels were evaluated using CAG-IMR for coronary microvascular dysfunction (CMD) detection, utilizing wire-based IMR as the reference standard. CMD was determined based on wire-based IMR ≥25U. CAG-IMR was independently calculated from diagnostic coronary angiography in a blinded fashion, employing the same diagnostic threshold of 25U for CMD identification.

**Results:**

CAG-IMR demonstrated significant correlation (*r* = 0.84, *p* < 0.001) and good diagnostic performance AUC = 0.97 (95% CI: 0.95–0.99) compared to wire-based IMR. It exhibited the overall diagnostic accuracy at 95.0% (95% CI: 92.0%−98.0%), alongside high sensitivity (92.7%) and specificity (95.6%). The positive predictive value (PPV) stood at 84.4%, and the negative predictive value (NPV) reached 98.1%.

**Conclusions:**

This study introduces CAG-IMR, a novel, multi-branch and wire-free method for IMR calculation. The indicator demonstrates good diagnostic accuracy and correlation with wire-based IMR in a cohort of 139 patients and 201 vessels, with the potential to enhance clinical CMD assessment.

## Introduction

Over the last two decades, numerous studies have reported the occurrence of coronary microvascular dysfunction (CMD) in various clinical conditions that lead to myocardial ischemia (1). CMD refers to an abnormality in the structure and function of coronary microcirculation, which affects coronary blood flow and oxygen supply to myocardial tissue, leading to a clinical syndrome characterized by exertional angina or myocardial ischemia (2). Patients with CMD are usually associated with ischemia and no obstructive coronary artery disease (INOCA), which plays a pivotal role in cases of angina pectoris. Angina pectoris affects ~112 million people worldwide (3). A large proportion of patients (up to 70%) undergoing coronary angiography because of angina and evidence of myocardial ischemia do not have obstructive coronary arteries but have demonstrable ischemia (4). Among these patients, a significant number experience INOCA as the primary cause of their symptoms (5), with INOCA being largely linked to CMD (6, 7). Due to the diminutive size of coronary microcirculation, it eludes visualization through both invasive and non-invasive coronary angiography. Consequently, the diagnosis of CMD pivots on the functional assessment of coronary arteries (8).

Currently, one of the primary indicators for assessing CMD is the wire-based index of microcirculatory resistance (IMR) in clinical practice (9). Wire-based IMR is calculated as the ratio of the pressure, *P*_*d*_, at the distal end of the coronary artery during hyperemia to the reciprocal of the transit time. The wire-based IMR serves as indicator for hyperemic microvascular resistance in coronary artery. Its value lies in providing crucial insights into predicting, diagnosing, treating, and prognosticating CMD. The advantages of wire-based IMR include its quantitative assessment of microcirculatory resistance, consistent measurement reproducibility, and independence from hemodynamic fluctuations, among other benefits (10). Recent research suggests that wire-based IMR plays a significant role in diagnosing microcirculatory disorders (11) and is able to identify patients at risk of long-term major adverse cardiac events (MACE) (12, 13). To date, wire-based IMR is considered as one of most important tools for assessing CMD, especially in those patients with Takotsubo syndrome (TTS), INOCA and ANOCA (11, 14, 15) in which CMD might serve as significant contributing mechanism.

However, wire-based IMR necessitates the use of pressure wires to measure pressure and employs thermodilution techniques to assess transit time. This entire process is intricate, time-consuming, costly and sometime it may cause lesion like coronary artery dissection (16, 17). Additionally, there is a requirement for drug-induced maximal hyperemia during pressure measure procedure, which poses the risk of some significant side effects to patients (18).

These limitations restrict the application of wire-based IMR in routine clinical practice. To overcome these challenges, researchers have recently explored pressure-wire-free methods based on angiography, leading to the development of angiography-based IMR (19). The previously proposed angiography-based IMR methods (20–22) have demonstrated a relatively high level of diagnostic accuracy (~80%) for CMD.

In this study, we used an innovative approach with a multi-branch patient-specific algorithm implemented in a dedicated software system (AngioQFA, Raysight Medical, Shenzhen, China) to calculate the coronary angiography-derived IMR (CAG-IMR). This study therefore sought to assess the clinical diagnostic performance of CAG-IMR using wire-based IMR as the reference standard.

## Materials and methods

### Study design

This was a single-center, retrospective, and observational study. The study aimed to assess the diagnostic performance of the innovative and multi-branch CAG-IMR in detecting clinically significant coronary microvascular dysfunction, using wire-based IMR as the reference standard.

The retrospective study received approval from the Institutional Review Boards of Hospital [No. 2022150K], Wuhan University, China, in compliance with the Declaration of Helsinki and the Good Clinical Practice Guidelines established by the China National Medical Products Administration. Written consent for the anonymous collection of data was obtained from all participating patients.

### Study population

In this retrospective study, patients who were suspected with coronary artery disease and underwent invasive coronary angiography (ICA) and wire-based IMR measurement between March 2011 and October 2017 at ZhongNan Hospital of Wuhan University (Wuhan, China) were considered.

Patients meeting any of the following criteria were excluded: (a) left ventricular ejection fraction (LVEF) ≤ 50%, (b) estimated glomerular filtration rate (eGFR) < 45 mL/min/1.73 m^2^, (c) severe coagulopathy or bleeding disorders, and (d) allergy to iodine contrast agents or vasodilators. Angiographic exclusion criteria included: (a) angiographic projections separated by < 25°, (b) unanalyzable poor image quality, (c) poor contrast opacification, (d) unsatisfactory projection view, (e) severe overlap or distortion of the target vessel, (f) frame rate ≤ 7.5/s.

A total of 192 patients were initially identified before employing the exclusion criteria for patients. Nineteen patients were excluded due to missing angiographic image, missing target vessel information, or LVEF ≤ 50%. In addition, 35 patients were excluded for the angiographic exclusion criteria including close image angle (*n* = 8), severe overlap or distortion (*n* = 14), poor contrast opacification (*n* = 12). In the end, this study included a total of 139 patients with 201 vessels who underwent both wire-based IMR measurement and CAG-IMR calculation.

### CAG-IMR calculation

The computational process of CAG-IMR primarily comprises model reconstruction and computational fluid dynamics (CFD) simulation. Model reconstruction involves the utilization of two angiographic images at rest with angiographic projections separated by ≥25°. Through the application of a deep learning neural network architecture, the blood vessel centerlines and contours are extracted. The operator can manually modify the contour boundary if there are any mismatch between the automated extracting contour line and real vessel boundary. Ultimately, a three-dimensional (3D) reconstructed vascular model with multiple branches is achieved, by leveraging the 3D spatial relationship of the vessels and their corresponding branches.

**Figure 1:**
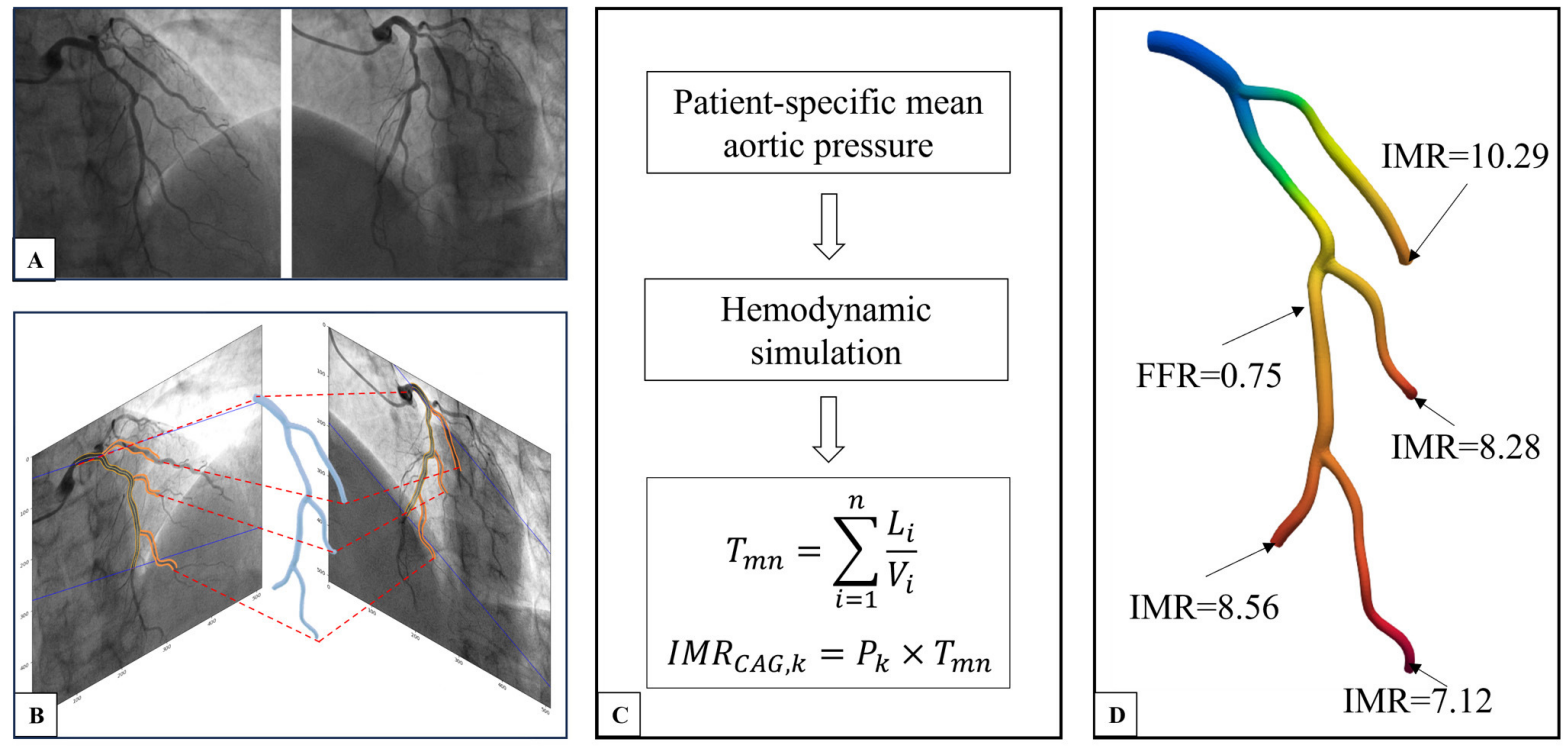
CAG-IMR calculation method flow chart. **(A)** Coronary angiogram image of the target vessel. **(B)** Three-dimensional reconstruction of the multi-branch coronary artery tree. **(C)** Acquiring patient-specific mean aortic pressure, conducting hemodynamic simulation, and calculating CAG-IMR. **(D)** Post-processing of results to obtain CAG-FFR in the entire coronary tree and CAG-IMR of each branch. CAG, Coronary Angiography; IMR, Index of Microcirculatory Resistance; FFR, Fractional Flow Reserve.

**Figure 2:**
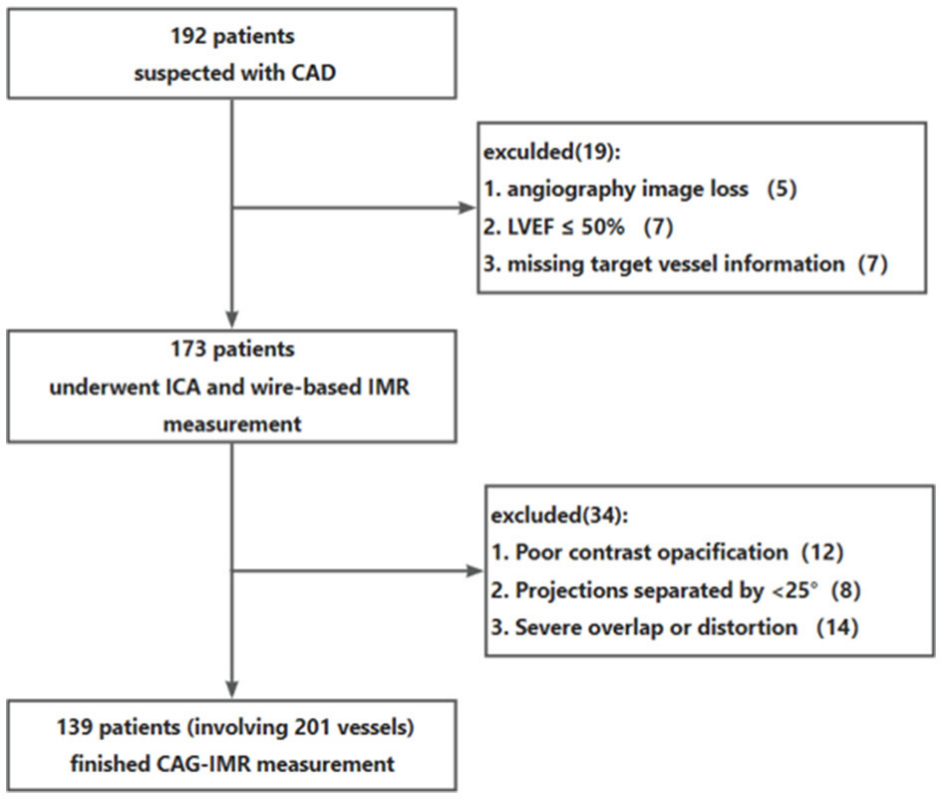
Patients flow chart. CAD, coronary artery disease; ICA, invasive coronary angiography; IMR, index of microcirculatory resistance.

Upon obtaining the vascular model, a CFD approach based on patient-specific boundary conditions is utilized to calculate the flow equations. The patient-specific boundary conditions include inlet flow rate and inlet pressure. Inlet flow rate was determined by vascular volume and flow time deriving from contrast through TIMI frame count, which can be done manually. Inlet pressure was set to be patient-specific mean aortic pressure acquired from measuring process (Figures 1, 2).

The coronary artery was divided into segments in order to set reasonable flow rate along the main vessel as some daughter branches which might not be included in the 3D model because of too small size or poor image quality split the flow in the in practice. The flow rate of each segment decreases along the vessel according to the reference diameter, with the method of determining reference diameter derived from previous study (23). Given the above patient-specific boundary conditions, the CFD simulation computes the velocity and pressure at various positions of the multi-branch coronary arteries. For the *k*_*th*_ branch, the index of microcirculatory resistance can be approximated as follow


$$\begin{array}{l} IMR_{CAG,\;k}=P_{k}\times T_{mn} \end{array}$$

where *P*_*k*_ represents the distal pressure of the *k*_*th*_ branch, *T*_*mn*_ represents the transit time defined as


$$\begin{array}{l} T_{mn}=\overset{n}{\underset{i=1}{\sum }}\frac{L_{i}}{V_{i}} \end{array}$$

Where *i* represents the *i*_*th*_ vascular segment, *L* denotes the length of the segment, and *V* represents the blood flow velocity at that segment, *n* represents the total number of segments counting from the proximal segment of the primary coronary artery inlet extending to the distal segment of the *k*_*th*_ branch. By cumulatively summing the flow transit time for each vascular branch, we can obtain a more precise *T*_*mn*_.

**Figure 3:**
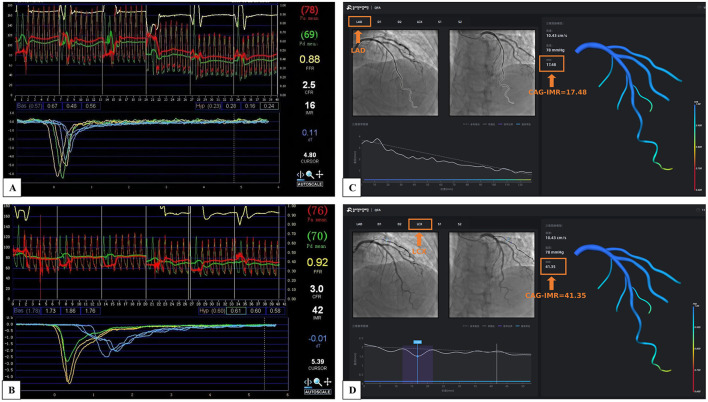
The multi-branch coronary artery tree CAG-IMR results of a single patient. **(A)** Wire-based IMR at LAD. **(B)** Wire-based IMR at LCX. **(C)** CAG-IMR at LAD. **(D)** CAG-IMR at LCX.

Figure 3 illustrates a patient with CMD in two vessels (LAD and LCX). In Figures 3A, B, clinical measurements of wire-based IMR confirm that both LAD and LCX IMR values is larger than 25U. Figures 3C, D present CAG-IMR results, yielding positive CMD diagnoses consistent with wire-based IMR. CAG-IMR, as a multi-branch method, provides a significant advantage by enabling the assessment of microcirculatory function in multiple vessel branches in a single computation.

The analysis process of CAG-IMR involves angiography image processing, model reconstruction, three-dimensional computational fluid dynamics (CFD) simulation, and post-processing of results. Thanks to the application of deep learning in angiography image processing and optimization of the CFD algorithm, the entire calculation process can be completed on a personal computer within 5 min.

### Wire-based IMR measurement

The wire-based IMR was determined using the established thermodilution technique with a pressure wire from St. Jude Medical (St. Paul, MN, United States). The procedures followed the standard guidelines recommended by the RadiAnalyzer Xpress instrument, also from St. Jude Medical. The sensor on pressure wire was first calibrated and equalized to ensure accuracy, then the pressure wire was positioned distally to the target vessel. Prior to commencing physiological measurements, an intracoronary administration of 100 μg of nitroglycerine was performed to prevent potential spasms. A controlled intravenous administration of adenosine at a rate of 140 μg/kg/min was carried out to induce hyperemia. The aortic pressure (Pa) and distal pressure (Pd) as well as the measurement site were recorded once the drug worked and hyperemia reached maximal and stable state. During sustained hyperemia, the mean transit time (Tmn) was computed as the average of transit time measurements obtained during three separate injections of 3–4 mL of room-temperature saline. Wire-based IMR could be calculated by the following equation (24):


$$\begin{array}{l} IMR_{wire}=P_{d}\times T_{mn} \end{array}$$

After the measurement, the pressure wire was meticulously pull-back to the guiding catheter tip to avoid any large potential pressure drift. The allowable range for the ratio of wire-based mean pressure to mean arterial pressure (MAP) is set between 0.97 and 1.03.

### Reproducibility and statistical analysis

Continuous variables were summarized as mean ± standard deviation or median with 25th and 75th percentiles, while categorical variables were presented as counts and percentages. Spearman's correlation coefficient examined relationships between variables, and linear regression analysis quantified these associations. Bland-Altman analysis assessed agreement between variables and different operators. The current study takes wire-based IMR ≥25U as the reference standard to evaluate diagnostic performance of CAG-IMR via receiver-operating characteristic (ROC) curve analysis (5, 25). Inter-observer reproducibility of CAG-IMR calculations involved two independent operators analyzing 30 randomly selected vessels in a blinded manner. Additionally, intra-observer reproducibility of CAG-IMR calculations is conducted by one operator at the interval of 1 week. All analysis procedure must follow the same standard operation and being blinded to each other or the previous analysis results. Paired measurements were compared using correlation coefficients and paired sample *t*-tests. Significance was established at a *p*-value < 0.05, with all analyses conducted using Python. This comprehensive analytical approach ensured a robust assessment of the data.

## Results

### Baseline characteristics

Wire-based IMR and CAG-IMR were successfully obtained from a cohort of 139 patients with 201 coronary vessels involved. Baseline demographic and vessel-related characteristics were presented in Table 1. The predominant patient presentation is acute coronary syndromes (ACS) (61.1%), including unstable angina, ST-elevation myocardial infarction (STEMI) and Non-ST-elevation myocardial infarction (NSTEMI), while there were 24 patients (17.3%) for unknown causes or non-coronary relating disease. In the context of the target vessels, the left anterior descending (LAD) artery comprised 55.7%, while the left circumflex (LCX) artery accounted for 23.4%, and the right coronary artery (RCA) represented 20.0%. Among the cohort of 139 patients, approximately one-third of the patients underwent measurements of both wire-based IMR and CAG-IMR at two or three target vessels.

**Table 1:** Baseline characteristics.

**No. of patients**	***n* = 138**
**Age, years**	
Mean ± SD	62.63 ± 11.97
Range	21–82
Male, *n* (%)	85 (61.1)
Body mass index, kg/m^2^	23.58 ± 2.61
Systolic blood pressure, mmHg	133.84 ± 19.92
Diastolic blood pressure, mmHg	76.1 ± 10.69
Left ventricular ejection fraction, %	58.07 ± 7.43
Diabetes, *n* (%)	34 (24.5)
Hypertension, *n* (%)	82 (58.9)
Hyperlipidemia, *n* (%)	47 (33.8)
Current smoker, *n* (%)	45 (32.4)
Prior PCI, *n* (%)	9 (6.5)
Prior CABG, *n* (%)	0 (0)
Prior myocardial infarction, *n* (%)	13 (9.4)
Silent ischemia, *n* (%)	4 (2.9)
Stable angina pectoris, *n* (%)	24 (17.3)
Unstable angina pectoris, *n* (%)	35 (25.2)
Clinic diagnosis, *n* (%)	*N* = 139
ACS	85 (61.1)
CCS	30 (21.6)
Others	24 (17.3)
STEMI	36 (25.9)
UNSTEMI	15 (10.8)
**Target vessel**, ***n*** **(%**)	***N*** = **201**
LAD	112 (55.7)
LCX	47 (23.4)
RCA	40 (19.9)
Others	2 (1.0)
**Number of vessels**, ***n*** **(%)**	
One	89 (64.1)
Two	38 (7.3)
Three	12 (8.6)
**Parameter**, ***n*** **(%)**	
IMR>25U	38 (26.6)
Mean IMR	18.63 ± 7.65
Mean CAG-IMR	17.64 ± 8.71

Values are the mean ± SD or *n* (%). SD, standard deviation Values are the mean ± SD or % (n).

PCI, percutaneous coronary intervention; CABG, coronary artery bypass graft; LAD, left anterior descending artery; LCX, left circumflex artery; RCA, right coronary artery; IMR, index of microcirculatory resistance; ACS, Acute coronary syndromes; CCS, chronic coronary syndromes; STEMI, ST-elevation myocardial infarction; NSTEMI, Non-ST-elevation myocardial infarction; others, unknown cause or non-coronary relating disease.

### Correlation and diagnostic performance of CAG-IMR

**Figure 4:**
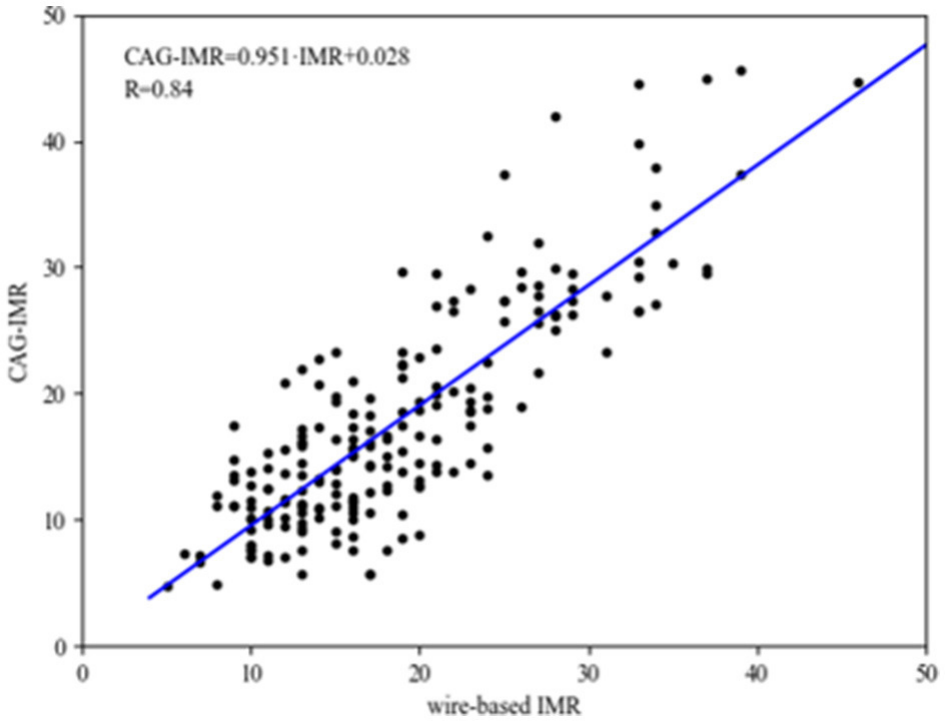
The correlation and linear regression analysis between CAG-IMR and wire-based IMR. The CAG-IMR values (depicted on the *y*-axis) exhibited a robust positive correlation (*r* = 0.84, *p* < 0.001) with wire-based IMR (illustrated on the *x*-axis).

**Figure 5:**
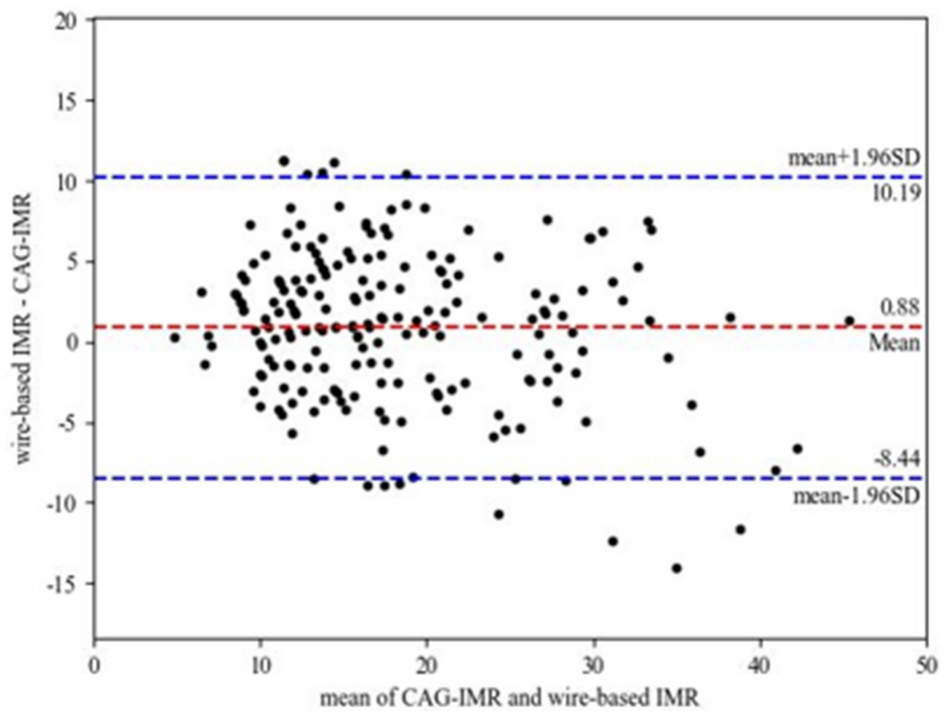
Bland-Altman plots for pairwise comparison (mean difference: 0.88; SD: 4.75; 95% limits of agreement −8.44 to 10.19).

Figure 4 shows the correlation and linear regression analysis performed to assess the relationship between CAG-IMR and wire-based IMR. CAG-IMR demonstrated a notably strong concordance with wire-based IMR, as indicated by a correlation coefficient of *r* = 0.84 with a statistically significant *p* < 0.001. Similarly, Figure 5 displays Bland-Altman plots comparing CAG-IMR and wire-based IMR, further confirming the high level of agreement. Notably, CAG-IMR exhibits a slight underestimation of IMR values when using wire-based IMR as the reference standard. The mean difference between the two measurements is 0.88 ± 4.75.

**Figure 6:**
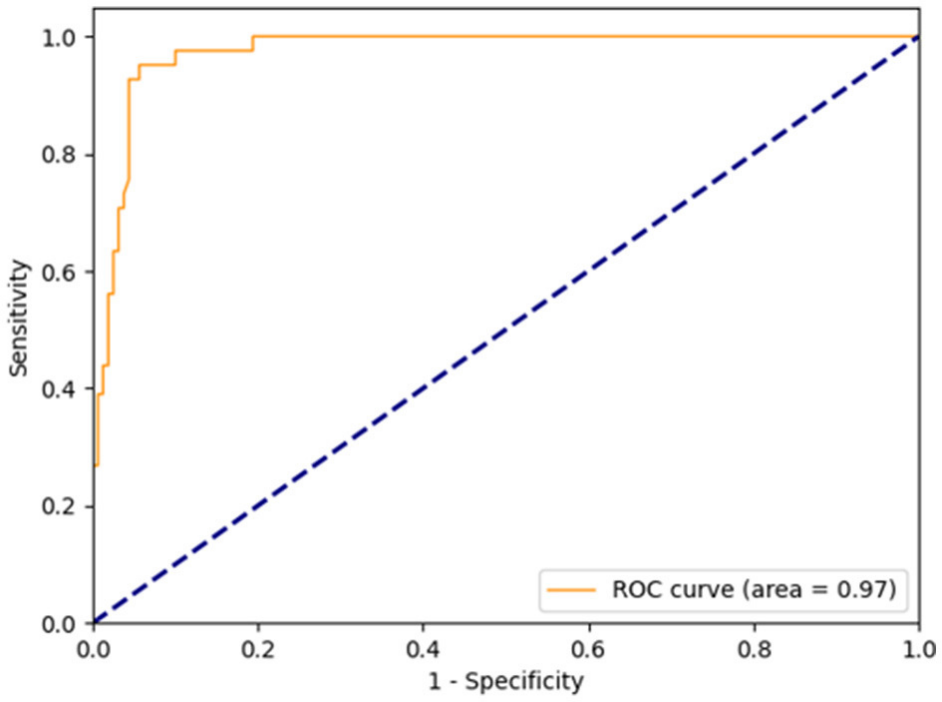
Receiver-operating curve for CAG-IMR, using a cutoff value of 25. The area under the curve (AUC) was calculated to be 0.97.

The diagnostic criteria for coronary microvascular dysfunction (CMD) were established as wire-based IMR ≥ 25U and CAG-IMR ≥ 25U in current study. As illustrated in Figure 6, the ROC curves were generated for CAG-IMR, employing a threshold value of 25U. The computed Area Under the Curve (AUC) yielded a value of 0.97 (95% CI: 0.95–0.99), signifying that CAG-IMR serves as a robust and effective method for CMD diagnosis.

The comprehensive evaluation of CAG-IMR revealed an impressive diagnostic performance with an overall diagnostic accuracy of 95.0% (95% CI: 92.0%−98.0%) as show in Table 2. Sensitivity and specificity were notably high, with values of 92.7% (95% CI: 84.7%−100.0%) and 95.6% (95% CI: 92.5%−98.8%), respectively. The positive predictive value (PPV) was 84.4% (95% CI: 73.9%−95.0%), and the negative predictive value (NPV) reached 98.1% (95% CI: 95.9%−100.0%). Of note, CAG-IMR showed numerically lower diagnostic accuracy in patients with chronic coronary syndromes (CCS) (88.1%, 95% CI: [78.3%−97.9%]) than ACS (96.7%, 95% CI: [93.5%−99.9%]) and the other group (97.4%, 95% CI: [92.5%−100.0%]). However, no statistically difference was found between CCS and ACS (*p* = 0.07) or CCS and other group (*p* = 0.4). In addition, there is no significant difference (*p* = 0.99) between the subgroups of STEMI (98.0%, 95% CI: [94.0%−100.0%]) and NSTEMI (90.0%, 95% CI: [76.9%−100.0%]) in patients with ACS. In terms of the main coronary arteries, the diagnostic performance is close for LAD, LCX and RCA with accuracy of (94.6%, 95% CI: [90.5%−98.8%]), (95.7.0%, 95% CI: [90.0%−100.0%]), (95.2%, 95% CI: [88.8%−100.0%]) (*P* = 0.63). Similarly, there is no statistic difference in the agreement of CAG-IMR and wire-based IMR in patients with obstructive CAD and those with non-obstructive CAD (95.9%, 95% CI: [91.5%−100.0%] vs. 94.5%, 95% CI: [90.5%−98.5%], *p* = 0.83) as in Table 3 show.

**Table 2:** Diagnostic performance of CAG-IMR by using wire-based IMR ≥25 as the reference standard.

**Diagnostic characteristic**	**ALL**	**ACS**	**CCS**	**Others**
Sensitivity, %	92.7 (84.7–100.0)	91.7 (80.6–100.0)	90.0 (71.4–100.0)	100.0 (100.0–100.0)
Specificity, %	95.6 (92.5–98.8)	97.89 (95.1–100.0)	87.5 (76.0–99.0)	96.9 (90.8–100.0)
PPV, %	84.4 (73.9–95.0)	91.7 (80.6–100.0)	69.2 (44.1–94.3)	87.5 (64.6–100.0)
NPV, %	98.1 (95.9–100.0)	97.9 (95.1–100.0)	96.6 (89.9–100.0)	100.0 (100.0–100.0)
Accuracy, %	95.0 (92.0–98.0)	96.7 (93.5–99.9)	88.1 (78.3–97.9)	97.4 (92.5–100.0)

**Table 3:** Diagnostic performance of CAG-IMR in subgroup by using wire-based IMR ≥25 as the reference standard.

**Diagnostic characteristic**	**Vessel**			**Obstructive**	**Non-obstructive**
	**LAD**	**LCX**	**RCA**		
Sensitivity, %	95.7 (87.3–100.0)	87.5 (64.6–100.0)	90.0 (71. 99–100.0)	90.0 (76.9–100.0)	95.2 (86.1–100.0)
Specificity, %	94.4 (89.6–99.2)	97.4 (92.5–100.0)	96.9 (90.8–100.0)	95.3 (91.3–99.3)	96.2 (91.1–100.0)
PPV, %	81.5 (66.8–96.1)	87.5 (64.6–100.0)	90.0 (71.4–100.0)	78.3 (61.4–95.1)	90.9 (78.9–100.0)
NPV, %	98.8 (96.5–100.0)	97.4 (92.5–100.0)	96.9 (90.8–100.0)	98.1 (95.4–100.0)	98.1 (94.3–100.0)
Accuracy, %	94.6 (90.5–98.8)	95.7 (90.0–100.0)	95.2 (88.8–100.0)	94.5 (90.5–98.5)	95.9 (91.5–100.0)

### Reproducibility and computational performance of CAG-IMR analysis

**Figure 7:**
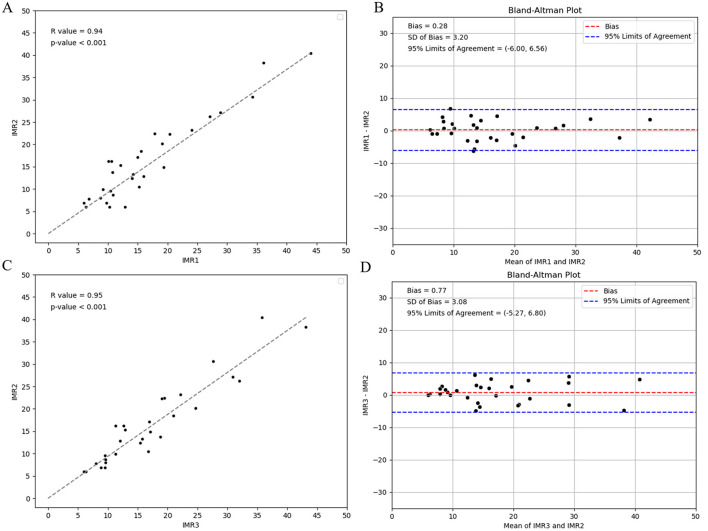
Intra-observer reproducibility analysis of CAG-IMR calculations by one operator at the intervals of 1 week and Inter-observer reproducibility analysis by two operators: **(A)** Assessment of consistency in CAG-IMR calculations, **(B)** Bland-Altman plot illustrating agreement. Inter-observer reproducibility analysis of CAG-IMR calculations: **(C)** Evaluation of consistency between two sets of CAG-IMR calculations, **(D)** Bland-Altman plot demonstrating agreement.

Figure 7 presents an evaluation of the reproducibility of CAG-IMR analysis, encompassing both intra-observer and inter-observer assessments. The observed agreements in both cases exhibit high consistency, with correlation coefficients of *r* = 0.94 (*p* < 0.001) for intra-observer assessments and *r* = 0.95 (*p* < 0.001) for inter-observer assessments, underscoring the robust consistency and reliability of the operated process in AngioQFA.

## Discussion

This study primarily focused on the diagnostic performance of CAG-IMR in a retrospective single-center patient cohort, employing wire-based IMR results as the reference standard. The findings underscore a good correlation (*r* = 0.84) between CAG-IMR and wire-based IMR, with CAG-IMR demonstrating a high diagnostic accuracy of 95.0% when using threshold of IMR ≥ 25U to define coronary microvascular dysfunction (CMD) positivity. Furthermore, the AUC reached to 0.97, highlighting the robust diagnostic capability of CAG-IMR in CMD assessment.

Various methods have emerged to assess ischemia in CMD with advancements of coronary artery diagnostic technologies, encompassing both non-invasive and invasive approaches. Numerous non-invasive techniques such as cardiac magnetic resonance (CMR), positron emission tomography (PET), magnetic resonance imaging (MRI), and single-photon emission computed tomography (SPECT) have been utilized to identify ischemia in CMD (2). However, these techniques usually entail high costs, complicated procedures and potential radiation hazards. In comparison, CAG-IMR stands as a novel angiography-based IMR method. It demands less costly equipment investment and derives IMR from invasive coronary angiography without drug-induced hyperemia and additional radiation, consequently reducing potential harm to patients.

Conversely, invasive metrics for assessment of CMD include coronary flow reserve (CFR) and IMR that can be acquired by the method of bolus thermodilution or continuous thermodilution and intracoronary Doppler (26–29). These two indices have gained significant recognition and, to some extent, have been applied in clinical practice. The 2019 ESC CCS guidelines (30) have granted a Class IIa recommendation for wire-based measurement of CFR and/or IMR in patients with persistent symptoms, yet possessing coronary arteries that are either angiographically normal or have moderate stenoses with preserved instantaneous wave-free ratio (iwFR) or fractional flow reserve (FFR). CFR denotes the ratio of coronary artery blood flow rate during maximal hyperemia to the corresponding values at rest, offering a comprehensive gauge of the coronary artery system's reserve capacity. Therefore, factors involving both epicardial coronary arteries and microvascular influences can impact CFR values, thus the presence of severe obstructive disease in the epicardial arteries must be ruled out to diagnose CMD using CFR. Consequently, CFR lacks specificity in detecting microcirculatory changes (9). Another novel marker is microvascular resistance reserve (MRR), which was proposed recently as a specific, quantitative, and operator-independent metric to quantify CMD (31, 32). An MRR value about 2.7–3 may be considered normal (33, 34), however, to fully validate its application in clinical settings, additional researches are necessary to ensure its efficacy and reliability in real-world scenarios.

Unlike CFR, wire-based IMR could serve as a reliable tool for evaluating the condition of the microcirculation. Importantly, the utility of IMR extends beyond the diagnostic phase. For example, IMR can assist to determine whether microvascular dysfunction is the primary cause of chest pain in cases of non-obstructive coronary disease. Additionally, in stable patients undergoing percutaneous coronary intervention, IMR is able to act as indicator to predict the risk of periprocedural infarction. This information, in turn, guides additional prevention measures and helps determine the appropriate course of treatment.

The main challenges limiting the clinical routine use of wire-based IMR include (a) inducing maximal hyperemia by using adenosine is required, especially in patients who are allergic to adenosine, (b) the use of pressure wires in patients without coronary stenosis, and (c) the extension of procedural time (21).

In response to challenges associated with wire-based IMR, multiple pressure-wire-free IMR methodologies based on angiography have emerged recently (20, 35–37). These methodologies employ diverse approaches to evaluate maximal hyperemia without using adenosine and utilize 3D or reduced-order models to solve equations for pressure and velocity, ultimately calculating angiography-based IMR. These approaches have demonstrated relatively good diagnostic performance in various conditions such as STEMI (37), acute coronary syndrome, chronic coronary syndrome (38), and among others (19, 22).

However, the vascular models utilized in these methodologies primarily consist of single-vessel models without daughter branches (16, 35). Reconstructing algorithms for single-vessel models is a straightforward process that requires less computational simulation for hemodynamics. However, these models lack crucial information about branch vessels, including their sizes and positions. Consequently, single-vessel models without branches cannot precisely estimate flow distribution at bifurcations, leading to potential errors in calculations. Indeed, these methodologies face challenges in accurately evaluating patients with near occlusion or bifurcation lesions due to the absence of the collateral and side branches in the models.

To address issues related to single-vessel angiography-based IMR, this study introduced CAG-IMR, which is an innovative, multi-branch methodology designed for IMR calculation. This CAG-IMR utilizes a multi-branch algorithm supported by deep learning enabling the segmentation and reconstruction of complex, branching 3D vascular models. By employing patient-specific boundary conditions and solving fluid dynamic equations, CAG-IMR could be determined for each vascular branch. Notably, one time computation of CAG-IMR enables the simultaneous determination of multiple critical microcirculatory indices, eliminating the necessity for multiple simulations when evaluating patients with multiple vascular branches. For instance, when estimating IMR values in patients with left main coronary artery disease, CAG-IMR allows for the concurrent assessment of microcirculatory function at the distal ends of the left anterior descending (LAD) and left circumflex (LCX) arteries. Furthermore, in patients with bifurcation lesions, it enables the simultaneous evaluation of microcirculatory function in both the main branch and the side branch.

The results in this study indicate that CAG-IMR's diagnostic accuracy is comparable with the most recent angiography-based IMR methods (19, 38). In the subgroup analysis, the CAG-IMR showed lower diagnostic accuracy in patients with chronic coronary syndromes than ACS. As no significant difference was found between these two groups, the reason might be sample size difference. There are only 42 vessels in CCS group, while 120 vessels in ACS group. The results also show CAG-IMR performed almost equally among different vessels, indicating its significant clinical meaning. Furthermore, previous literature showed that CMD is a factor of FFR/non-hyperemic index discrepancy (39), this non-invasive index is promising in improving the performance of angiography derived FFR as it can provide more precise resistance, which is the key boundary condition for CFD.

### Limitations

Firstly, this study is a retrospective, single-center investigation, which has certain limitations regarding its representation of geographic and ethnic diversity. Nonetheless, it is noteworthy that the study included a relatively large cohort of 139 patients, making it one of the studies with a substantial number of participants in the field of IMR-related research. Second, there are some differences between the values of wire-based IMR and CAG-IMR, potentially stemming from the empirical equation used to estimate hyperemia in the calculation of CAG-IMR, while hyperemia is drug-induced in wire-based IMR measurements. Third, further research is required to compare the multi-branch method with the single-vessel method in evaluating microvascular function at the distal ends of multiple coronary vessels in patients. Lastly, boundary condition plays a crucial role in simulations, as different inflow boundary conditions may significantly impact the result (40), thus there is a pressing need for more precise inflow boundary conditions in future studies.

## Conclusions

This study introduces CAG-IMR, an innovative, multi-branch methodology with patient-specific boundary conditions for calculating the IMR based on angiography. In contrast to the conventional wire-based IMR measurement, CAG-IMR represents a wire-free methodology that offers several advantages, including time efficiency, cost reduction, and alleviation of patient discomfort when evaluating coronary microvascular dysfunction. In a cohort of 139 patients and 201 vessels, CAG-IMR demonstrated a significantly elevated degree of diagnostic accuracy and a strong correlation when compared to wire-based IMR, used as the reference standard. In conclusion, CAG-IMR holds the potential to emerge as a valuable diagnostic tool for the routine clinical assessment of CMD.

## Data Availability

The original contributions presented in the study are included in the article/supplementary material, further inquiries can be directed to the corresponding authors.
