# Corrigendum: Diagnostic performance of multi-branch coronary angiography-based index of microcirculatory resistance: a novel approach

**DOI:** 10.3389/fmed.2025.1565358

**Published:** 2025-04-09

**Authors:** Yongzhen Fan, Shuang Wang, Xinyong Cai, Xiaorong Hu, Jun Ma, Hongzhi Lan, Zhibing Lu

**Affiliations:** ^1^Institute of Myocardial Injury and Repair, Wuhan University, Wuhan, China; ^2^Department of Cardiology, Zhongnan Hospital, Wuhan University, Wuhan, Hebei Province, China; ^3^Department of Cardiovascular Ultrasound, Zhongnan Hospital of Wuhan University, Wuhan, China; ^4^Department of Cardiology, The First Affiliated Hospital of Nanchang Medical College, Nanchang, China; ^5^Shenzhen Raysightmed Co, Ltd, Shenzhen, China

**Keywords:** index of microcirculatory resistance (IMR), retrospective, coronary angiography-based IMR (CAG-IMR), computational fluid dynamics (CFD), coronary microvascular dysfunction (CMD)

In the published article, there was an error in the author list. The order of authors was erroneously given as:

Yongzhen Fan^1, 2^, Shuang Wang^3^, Xinyong Cai^4^, Zhibing Lu^1, 2*^, Jun Ma^5^, Hongzhi Lan^5*^ and Xiaorong Hu^1, 2^

The correct author list reads:

Yongzhen Fan^1, 2^, Shuang Wang^3^, Xinyong Cai^4^, Xiaorong Hu^1, 2^, Jun Ma^5^, Hongzhi Lan^5*^, Zhibing Lu^1, 2*^

The authors apologize for this error and state that this does not change the scientific conclusions of the article in any way. The original article has been updated.

